# Noonan Syndrome: Relation of Genotype to Cardiovascular Phenotype—A Multi-Center Retrospective Study

**DOI:** 10.3390/genes15111463

**Published:** 2024-11-13

**Authors:** Nikola Ilic, Stasa Krasic, Nina Maric, Vladimir Gasic, Jovana Krstic, Dimitrije Cvetkovic, Vesna Miljkovic, Boris Zec, Ales Maver, Vladislav Vukomanovic, Adrijan Sarajlija

**Affiliations:** 1 Clinical Genetics Outpatient Clinic, Mother and Child Health Care Institute of Serbia “Dr Vukan Cupic”, 11070 Belgrade, Serbia; ilicnikola91@gmail.com (N.I.); jovanakrst98@gmail.com (J.K.); 2 Department of Cardiology, Mother and Child Health Care Institute of Serbia “Dr Vukan Cupic”, 11070 Belgrade, Serbia; stasakrasic5@gmail.com (S.K.); vvladavuk64@gmail.com (V.V.); 3 Department of Pediatrics, Faculty of Medicine, University of Belgrade, 11000 Belgrade, Serbia; 4 Clinic for Children’s Disease, University Clinical Center of the Republic of Srpska, 78000 Banja Luka, Bosnia and Herzegovina; ninamaric.bl@gmail.com; 5 Laboratory for Molecular Biomedicine, Institute of Molecular Genetics and Genetic Engineering, University of Belgrade, 11042 Belgrade, Serbia; vlada.gasic42@gmail.com; 6 Department of Endocrinology, Mother and Child Health Care Institute of Serbia “Dr Vukan Cupic”, 11070 Belgrade, Serbia; dimitrije.cvetkovic@imd.org.rs; 7 Department of Endocrinology, University Clinical Center of the Republic of Srpska, 78000 Banja Luka, Bosnia and Herzegovina; vesnafd@gmail.com; 8 Department of Cardiology, University Clinical Center of the Republic of Srpska, 78000 Banja Luka, Bosnia and Herzegovina; zecboris75@gmail.com; 9 Clinical Institute of Genomic Medicine, University Medical Centre Ljubljana, 1000 Ljubljana, Slovenia; ales.maver@kclj.si

**Keywords:** Noonan syndrome (NS), cardio genetics, pediatric cardiology, MEK inhibitors

## Abstract

**Background:** Noonan syndrome (NS) is a congenital genetic disorder with a prevalence of 1 in 1000 to 2500 live births, and is characterized by distinctive facial features, short stature, chest deformities, and congenital heart disease. This study aims to evaluate the prevalence of specific genetic mutations and their impact on cardiovascular and other outcomes in NS. **Methods:** We conducted a retrospective clinical study of 25 pediatric patients diagnosed with NS at two institutions: The Mother and Child Health Care Institute of Serbia and the Clinic for Children Diseases, University Clinical Center of the Republic of Srpska. Patients underwent whole-exome sequencing (WES) to identify genetic mutations. Clinical data, including cardiovascular manifestations, psychomotor development, and stature, were analyzed in relation to mutation types. **Results:** The cohort comprised 60% male and 40% female patients, with a median age at diagnosis of 7.2 years. Cardiovascular abnormalities were present in 88% of patients. Mutations in *PTPN11* were most commonly associated with pulmonary valve stenosis (PVS), while *RAF1* mutations were prevalent in patients with hypertrophic cardiomyopathy (HCM). No significant association was found between cardiac disease and delayed psychomotor development (*p* = 0.755), even though the likelihood ratio showed significance in that regard (*p* = 0.018). Short stature was observed in 48% of patients but was not significantly correlated with genetic type of disease, presence of cardiac disease, or developmental delay. **Conclusions:** The study confirms the high prevalence of cardiovascular manifestations in NS and highlights genotype–phenotype correlations. While cardiac abnormalities are common, their impact on psychomotor development and stature is less clear. Further research is needed to explore genetic interactions influencing these outcomes and refine clinical management strategies.

## 1. Introduction

The Noonan syndrome (NS) is a relatively common congenital genetic disorder, with a prevalence of 1 in 1000 to 1 in 2500 live births [1]. It is characterized primarily by distinctive facial features, short stature, chest deformities, and congenital heart disease. NS follows an autosomal dominant inheritance pattern, showing complete penetrance but variable expressivity, which accounts for its wide range of clinical manifestations [2].

Dr. Jacqueline Noonan first described the syndrome in 1962 when she identified nine patients with shared facial characteristics, short stature, chest deformities, and pulmonary stenosis. The condition was later named in her honor after a 1968 case series further defined the syndrome [3]. The facial features of NS, such as hypertelorism, ptosis, and low-set ears, are most noticeable during infancy and early childhood. Over time, these features may become less pronounced, although certain characteristics, like ptosis and hypertelorism, can persist into adulthood [4]. NS also presents with various musculoskeletal abnormalities, including pectus deformities, scoliosis, and spinal anomalies [2]. Cardiovascular anomalies, most notably pulmonary valve stenosis (PVS), are common characteristics of the syndrome, along with other heart defects like hypertrophic cardiomyopathy (HCM) and atrial septal defects (ASDs) [5,6].

### 1.1. Genetic Mutations and Pathophysiological Mechanisms

Genetic research has significantly advanced the understanding of NS, linking it to mutations in the Ras/MAPK pathway, a critical regulator of cell growth and differentiation. These mutations are found in genes such as *PTPN11*, *SOS1*, *RAF1*, and *KRAS*, which account for approximately 60% of NS cases [7,8]. Despite these discoveries, clinical diagnosis remains essential, especially in cases without identified mutations [9].

The NS pathogenesis stems from disruptions in the Ras/MAPK pathway, leading to hyperactivation of cellular processes that control growth, proliferation, and differentiation [1]. This dysregulation explains the broad spectrum of clinical manifestations, including short stature, heart defects, and facial dysmorphisms [10]. *PTPN11* mutations are the most prevalent, accounting for around 50% of NS cases. These mutations lead to gain-of-function effects in SHP-2, a protein that regulates several signaling pathways [11]. Studies suggest that *PTPN11* mutations drive increased cellular proliferation and altered differentiation, contributing to the phenotypic diversity seen in NS patients [12].

Mutations in *SOS1*, *RAF1*, and *KRAS* also contribute to the pathogenesis of NS [13]. *SOS1* mutations are seen in 10–13% of cases and often lead to milder phenotypes compared to *PTPN11* mutations, while *RAF1* mutations, found in 3–17% of patients, are strongly associated with HCM [14]. *KRAS* mutations, though rarer, often result in a more severe phenotype, with significant developmental delay and cognitive impairment [15]. Less commonly, mutations in *NRAS*, *BRAF*, and *MAP2K1* have been linked to NS, all of which led to dysregulation of the Ras/MAPK pathway [16].

### 1.2. Cardiovascular Manifestations

Cardiovascular defects are a prominent feature of NS, with PVS being the most common. The narrowing of the right ventricular outflow tract in PVS often leads to right ventricular hypertrophy [16]. Other congenital heart defects, such as ASD and VSD, can increase the risk of heart failure due to abnormal blood flow patterns [17]. The most significant form of cardiomyopathy in NS is HCM, and it is driven mostly by *RAF1* and *RIT1* mutations that promote abnormal cardiac muscle growth through Ras/MAPK hyperactivation [16].

Recent advances in treatment have shown promise, and the use of MEK inhibitors like trametinib is of particular interest [18,19,20]. These drugs specifically target and inhibit the MEK1/2 enzymes, reducing the excessive signaling that drives pathological cardiac hypertrophy in RIT1-associated HCM [16]. Clinical studies have reported regression of myocardial hypertrophy and improved cardiac function in patients receiving trametinib, highlighting its potential as a targeted therapy for NS-related cardiac complications. MEK inhibitors also reduced nt-pro-BNP levels, a biomarker for heart failure, and improved key cardiac parameters like the left ventricular mass index [19,20].

This study aims to comprehensively evaluate the prevalence of specific genetic mutations associated with NS, identify potential genotype–phenotype correlations, and investigate their impact on cardiovascular manifestations as well as other clinical outcomes.

## 2. Materials and Methods

### 2.1. Study Design and Settings

Our research was designed as a retrospective study. From March 2014 to March 2024, we recruited 25 pediatric patients diagnosed with NS at the Mother and Child Health Care Institute of Serbia “Dr Vukan Čupić” in Belgrade, Serbia, and the Clinic for Children Diseases, University Clinical Center of the Republic of Srpska in Banja Luka, Republic of Srpska, Bosnia and Herzegovina.

The study received approval from the Institutional Ethics Committees of both centers (approval number 7060/2 and approval number 01-19-349-2/24). All patients underwent thorough clinical evaluation by clinical geneticists and a pediatric cardiologist.

Whole-exome sequencing (WES) was performed on all 25 patients at the Institute of Molecular Genetics and Genetic Engineering (IMGGE), Belgrade, Serbia, and the Clinical Institute of Medical Genetics at University Medical Ljubljana, Slovenia.

We collected and analyzed demographic and clinical parameters, as well as WES results, with an emphasis on the mode of inheritance; these data can be found in Table S1.

Informed consent for molecular diagnostics and further research use was obtained from the parents of all patients.

### 2.2. Data Curation

The data for this study were obtained from medical records, which included both paper and electronic formats, sourced from both centers.

Data cleaning involved removing duplicate entries and correcting any obvious data entry errors. We conducted a thorough review to ensure completeness and accuracy, with no missing data identified. Patient records were selected based on specific criteria, including the age (0–18 years), gender, and diagnosis of NS according to standardized assessment tools. We excluded patients with incomplete medical records and cases with clinical suspicion of NS without genetic confirmation to ensure sample homogeneity and minimize confounding factors.

### 2.3. Measurment

Anthropometric measurements were obtained using standardized techniques, including height measured with a stadiometer to the nearest 0.1 cm and weight recorded with a digital scale to the nearest 0.1 kg. Body mass index (BMI) was calculated as weight in kilograms divided by the square of height in meters (kg/m^2^).

Psychomotor development was assessed using standardized tools appropriate for each patient’s age. This standardized assessment was conducted at the time of diagnosis, employing specific developmental scales for different age groups (e.g., the Bayley Scales of Infant Development for ages 0–2, the Denver Developmental Screening Test for ages 2–5, and for older children, the Wechsler Preschool and Primary Scale of Intelligence or an equivalent scale). Additionally, anamnestic data on milestone achievements in verbal and gross motor development were analyzed for each patient. Due to the heterogeneity of age at diagnosis, psychomotor development was categorized as normal or delayed.

Echocardiographic measurements were performed using a high-frequency transducer suitable for pediatric patients. Key parameters included interventricular septal thickness (IVSd), left ventricular posterior wall thickness (LVPWd), and end-diastolic diameter (EDD). Z-scores for IVSd, LVPWd, and EDD were calculated based on age and body surface area-specific reference values, with the following formula:

Z = (observed value − mean reference value)/standard deviation.

We also assessed the presence of HCM and recorded the peak pressure gradients across the aortic and pulmonary valves.

DNA for WES analysis was isolated from peripheral blood samples using standard phenol–chloroform extraction and purification methods to ensure high-quality genomic material. Exome sequencing at the Institute of Molecular Genetics and Genetic Engineering, University of Belgrade, was performed using the Exome 2.0 Illumina panel (Illumina, Inc. San Diego, CA, USA) and compared to the reference human genome (hg38). The sequencing was conducted with the NextSeq2000 sequencer (Illumina, Inc. San Diego, CA, USA) and analyzed using Variant Interpreter software (The Illumina Variant Interpreter software v3.17), excluding variants in non-coding regions such as promoter and intronic areas, as well as repeat expansions.

Exome sequencing at the Clinical Institute of Genomic Medicine in Ljubljana utilized either the Illumina Nextera Rapid Capture Exome (Illumina, Inc. San Diego, CA, USA) or the Twist Core Exome (Twist Bioscience Corporation, South San Francisco, CA, USA). The Illumina Nextera Rapid Capture Exome covered 37 Mbp of the genome, while the Twist Core Exome covered 33 Mbp. Although mitochondrial sequences were not included in the capture during the exome-sequencing process, off-target reads were analyzed to evaluate mitochondrial DNA. This analysis was performed using specific bioinformatics tools designed to identify and quantify mitochondrial sequences from off-target reads. Sequencing was initially performed in the proband, and in cases where a potentially causative variant was detected, subsequent validation and segregation analysis using Sanger sequencing was performed on parental samples. The pathogenicity of the detected variants was classified according to the guidelines of the American College of Medical Genetics and Genomics and the Association for Molecular Pathology.

### 2.4. Statistical Analysis

Data were processed using statistical software SPSS 25.0 (IBM Corp., Armonk, NY, USA). Among the descriptive statistical methods, measures of central tendency (arithmetic mean, median, mode), measures of variability (standard deviation), and relative numbers (structure indicators) were used. The difference in the distribution of specific parameters among the studied groups was determined using χ^2^ or Fisher’s test. Shapiro–Wilk and Kolmogorov–Smirnov tests were used to assess the normality of the distribution of numerical variables. The paired *t*-test and Wilcoxon test were used to compare two dependent samples. All the statistical methods were considered statistically significant, *p* ≤ 0.05.

## 3. Results

In this study, we analyzed a cohort of 25 patients diagnosed with NS, of whom 60% were male and 40% female (Table 1).

Detailed clinical manifestations and organ involvement in patients with confirmed genetic causes of NS are presented in Table S1.

At the time of the study, 24 out of 25 patients were alive, with 1 patient lost to follow-up. The median age at genetic diagnosis was 7.2 years, ranging from 6m to 17 years. The most common subtype was type 1 (*PTPN11*), which was present in 48% of the patients, followed by type 5 (*RAF1*) in 20%. Regarding mutation pathogenicity, 76% of the mutations were classified as pathogenic, 16% as likely pathogenic, and 8% as variants of uncertain significance (VUS).

Cardiac manifestations were registered in 88% of patients. The most common cardiac manifestation was HCM (12/25), followed by pulmonary valve stenosis (9/25). Three patients had an ASD and one had VSD. One patient had an adult type of aortic coarctation (CoA), and he underwent surgical correction at the age of 2 months. Atrioventricular valve diseases were observed in five patients. Patients with ASD had *LZTR1*, *RIT1*, and *SOS1* mutations, while those with VSD had *KRAS* gene mutation. Two patients had supraventricular tachycardia—atrial ectopic tachycardia and antidromic reentrant tachycardia. Those patients had mutations in *RAF1* and *SOS1* genes. One patient with type (*RIT1*) had a prolonged QTc interval in the neonatal and early infant period.

The follow-up period from diagnosis to the last echocardiography examination was 1 year (IQR 0.5–1; min–max 0.5–5 years).

At the time of diagnosis, echocardiography findings are presented in Table 2.

Almost half (5/12) of the patients with HCM were referred to pediatric cardiologists due to a heart murmur. Ten male patients and two female patients had HCM. Type 1 (*PTPN11*) and type 5 (*RAF1*) were identified in five patients each, while no patients were diagnosed with type 2. All patients were heterozygotes. The most common mutation in the group of patients with HCM was observed in *RAF1* (5/12), followed by *PTPN11* (4/12), *RIT1* (1/12), *KRAS* (1/12), and *SOS1* (1/12). None of the patients with HCM had a mutation in *LRZT1* (Table 3). Six patients had the progressive form of HCM.

A Z-score of IVS diameter in diastole did not significantly change during follow-up (1.9; IQR 1.67–5.2 vs. 1.2; IQR 0.2–3.3; *p* = 0.4). Nine patients were treated with propranolol, and in one patient, trametinib was added due to rapidly progressive HCM and the development of a double-chamber right ventricle.

Eight out of ten patients with a PVS were referred to pediatric cardiologists due to a heart murmur. The first echocardiography examination revealed mild to moderate pulmonary stenosis (Table 1). PVS was less frequently observed in males (11/15 patients; *p* = 0.09). One patient was a combined heterozygote, while the rest were heterozygotes. Patients with type 1 NS (*PTPN11*) had the highest incidence of PVS (6 out of 10), while none of the patients with type 5 or type 3 NS exhibited a PVS.

Six out of ten patients with PVS had a mutation in *PTPN11*, two in *SOS1*, and one in *LTZR1* and *RIT1* (Table 1). In three patients with a *PTPN11* mutation, the same missense mutation, c.922A>G, was detected.

During the follow-up period, the pressure gradient across the pulmonary artery decreased slightly (29, IQR 20–38 mmHg) (Table 4).

Two patients underwent pulmonary balloon valvuloplasty (PBVP) due to progressive severe PVS. Both patients had a missense mutation in the *PTPN11*. The peak-to-peak gradient before balloon dilatation was 59 and 59 mmHg, while after balloon dilation, it was 18 mmHg. In a female patient, PBVP was performed twice, at ages 7 and 10. Four years after the second procedure, residual mild–moderate PVS lags were observed. In the male patient, mild PVS lags were noted.

Regarding psychomotor development, 36% of the patients exhibited developmental delay, while 64% had normal development. The chi-square analysis showed no statistically significant association between age at diagnosis groups and psychomotor delay (χ^2^ = 1.981, *p* = 0.739). The potential relation of specific genes associated with NS and psychomotor delay was determined by the chi-square test and showed borderline statistical significance (*p* = 0.054). The likelihood ratio test highlighted a significant result (*p* = 0.018), pointing to a potential influence of specific genes on psychomotor development. The chi-square test did not demonstrate a statistically significant association between the presence of cardiac disease and psychomotor development (*p* = 0.755).

In terms of stature, 48% of the patients had a z-score below −2 SD, although no significant association has been found between cardiac disease and stature (*p* = 0.920), nor between psychomotor development and stature (*p* = 0.494).

Analyses of the NS types revealed that neonatal cardiovascular manifestations appeared in approximately equal proportions, with 41.2% of patients with type 1 NS and 44.4% with type 5 NS (Table 5).

Heart murmurs were notably more frequent in type 1 patients; all 10 type 1 patients had murmurs, compared to only 8 patients with murmurs among other types.

The growth z-scores showed that half of NS patients with a *PTPN11* mutation had a short stature. Other NS types showed similar results, with the chi-square test confirming the absence of difference in stature between genetic types of NS (*p* = 0.260). HCM was observed in five type 1 patients, three type 5 patients, and three patients with other subtypes, while PVS has been recorded in five type 1 patients, two type 5 patients, and three others. In terms of psychomotor development, 6 type 1 patients had developmental delays compared to 11 with normal development, whereas other types had 5 patients with developmental delays and 10 with normal development. Lastly, the occurrence of other symptoms was slightly higher in type 1 patients, with six reporting additional symptoms, compared to five in other types (Table 6).

## 4. Discussion

This study highlights the significant cardiovascular manifestations in patients with NS, confirming their high prevalence and genetic basis. Almost 90% of our patients exhibited some form of cardiac involvement, with nearly half showing signs of HCM, a notably higher incidence than reported in other studies. Conversely, we observed a slightly lower incidence of PVS, contrasting with earlier reports that suggested that it occurs in nearly half of all patients with NS [3,6,21,22].

The increased frequency of HCM in our cohort can be attributed to several factors. First, the genetic variability within our patient population, particularly a higher prevalence of *RAF1* mutations known to correlate with HCM, likely contributes to this discrepancy. Additionally, the relatively small size of our cohort may introduce variability in observed outcomes and differences in diagnostic methods or screening protocols at various centers may also play a role in these variations.

A closer analysis of the genetic mutations in our cohort revealed specific genotype–phenotype correlations. *PTPN11* mutations were predominantly associated with PVS, while *RAF1* mutations showed a strong correlation with HCM, reflecting findings from previous reports [16,22,23]. This reinforces the role of the RAS/MAPK pathway in the pathogenesis of NS, with different mutations within the pathway leading to distinct cardiac manifestations. The relatively high frequency of HCM in patients with *RAF1* mutations further emphasizes the importance of early genetic screening and targeted surveillance for cardiomyopathy in this group [23].

HCM was most commonly observed in patients with *RAF1* mutations. Similarly to sarcomere-associated HCM, Noonan syndrome-associated HCM (NS-HCM) is characterized by fibrotic changes in the myocardium and myocyte disarray [24]. Unlike other forms of pediatric HCM, which typically manifest around the age of 8, NS-HCM often presents much earlier, with over half of the cases diagnosed by six months of age. Moreover, children with NS-HCM are at a higher risk of developing congestive heart failure compared to those with other types of HCM (24% versus 9%). They also tend to have more severe ventricular outflow obstruction, leading to higher rates of hospitalization and medical interventions [6,16]. This finding emphasizes the importance of early diagnosis and timely intervention.

Nearly half of the patients with HCM initially presented with a progressive form of the disease and were treated according to the HCM treatment guidelines, primarily with propranolol [25]. One of our patients exhibited very rapid progression of hypertrophy and developed a double-chambered right ventricle (DcRV), so we treated him with trametinib. Trametinib, a MEK 1/2 inhibitor, has shown favorable results in various studies addressing neurological, lymphatic, and cardiac manifestation [20,26,27,28,29,30,31,32].

PVS was the second most common cardiac manifestation in our cohort, with the most prevalent mutations detected in *PTPN11*. *PTPN11* encodes a protein tyrosine phosphatase called SHP-2. SHP-2 plays an important role in signal transduction for various biological processes, including forming semilunar valves [33]. The level of severity of PVS in patients with NS was similar to the reported rates [3]. None of our patients had critical PVS. However, two patients had severe PVS and required PBVP, with one of them needing re-intervention. The study conducted by Prendiville et al. found that three-quarters of patients who underwent initial PBVP still had clinically significant residual PVS, which required further intervention (either repeat PBVP or surgery). The risk factor for re-intervention was a younger median age at the time of the initial PBVP. Patients with NS are recognized to have dysplastic pulmonary valve leaflets that may inherently be more refractive to the relief of obstruction by PBPV [23].

Despite the clear cardiovascular involvement, our study did not demonstrate a statistically significant association between cardiac disease and psychomotor development. This lack of association suggests that while cardiac abnormalities are prevalent, they do not necessarily correlate with developmental outcomes. However, the likelihood ratio test hinted at potential genetic interactions affecting psychomotor development, a finding that warrants further exploration in larger cohorts [10].

Similarly, short stature emerged as a notable feature in our cohort, with nearly half of the patients presenting a stature below −2 SD. However, no significant association was found between growth impairment and cardiac disease or developmental delay. These results suggest that the growth impairment in NS is likely multifactorial, influenced by both genetic and possibly endocrine factors, rather than being directly linked to the cardiac manifestations [13,14,17].

The borderline statistical significance observed for the relation between specific genetic changes and psychomotor development suggests a possible genotype–phenotype correlation. This is particularly relevant given that certain mutations, such as those in *KRAS*, are known to be associated with more severe developmental delay phenotypes [14]. While our results did not reach full statistical significance, the trend observed aligns with existing literature, which highlights the importance of genotype in determining the severity of developmental outcomes in NS [12,14]. The lack of association between age at diagnosis and psychomotor delay may be attributed to genetic heterogeneity within the sample. Variations in genetic mutations could produce diverse phenotypic effects on psychomotor development, potentially masking any connection with the age at diagnosis. Additionally, the small cohort size further limits the statistical power to detect a meaningful association.

Our findings also highlight the importance of early diagnosis and genetic testing in NS, with the median age of diagnosis being slightly lower than that reported in previous studies. Early diagnosis is critical for the timely management of complications, particularly cardiovascular and developmental issues, which can significantly impact the patient’s quality of life [9,12,14,18].

## 5. Novel Contribution and Limitations of the Study

Although this study does not primarily aim to identify novel mutations associated with NS, it offers valuable insights into genotype–phenotype correlations, highlighting specific clinical features linked to known mutations. Additionally, while the cohort size may be considered small for a comprehensive analysis, the unique phenotypic and genotypic characteristics examined provide a distinct perspective that enhances our understanding of phenotypic variability and contributes to a more refined clinical characterization of NS.

## 6. Conclusions

This study reinforces the significant prevalence of cardiovascular manifestations in NS patients with the highest prevalence of PVS and HCM. Our findings confirm the role of specific genetic types of NS, particularly those associated with *PTPN11* and *RAF1* mutations, in shaping these cardiac abnormalities. No correlation was found between the presence of cardiac disease and delayed psychomotor development. Potential genotype–phenotype correlation was observed with regard to delayed development in NS patients. No risk factors for the short stature in NS were identified. Early clinical suspicion and final genetic diagnosis remain crucial for predicting complications and improving patient outcomes. Further research is needed to elucidate the complex genotype–phenotype relationships in NS and refine clinical management strategies.

## Figures and Tables

**Table 1:** Overview of genetic and cardiovascular findings in cohort.

Patients Number	Sex	Affected Gene	Initial CV S/S ^1^	Age of First CV S/S ^1^	Cardiovascular Manifestation
1	Female	*RAF1*	HM ^2^	Neonatal	HCM ^4^
2	Male	*RAF1*	HM ^2^	Neonatal	HCM ^4^
3	Female	*RAF1*	Chest pain, Fatigue	12 years	HCM ^4^
4	Female	*PTPN11*	HM ^2^	6 years	Physiological Murmur
5	Male	*PTPN11*	HM ^2^	Neonatal	HCM ^4^
6	Female	*PTPN11*	HM ^2^	Neonatal	PVS ^5^
7	Female	*PTPN11*	HM ^2^	2 months	PVS ^5^
8	Female	*PTPN11*	HM ^2^	Neonatal	PVS ^5^
9	Male	*PTPN11*	Fatigue, Hyperhidrosis	6 months	HCM ^4^
10	Male	*PTPN11*	None	N/A ^3^	None
11	Male	*SOS1*	HM ^2^	2 months	PVS ^5^
12	Female	*SOS1*	HM ^2^	2 years	PVS ^5^
13	Female	*SOS1*	Pericard Effusion	Neonatal	HCM ^4^
14	Male	*SOS1*	HM ^2^	Neonatal	PVS ^5^
15	Male	*LZTR1*	HM ^2^	Neonatal	PVS ^5^, VSD ^6^
16	Male	*KRAS*	HM ^2^	Neonatal	HCM ^4^
17	Male	*PTPN11*	HM ^2^	3 months	PVS ^5^
18	Male	*PTPN11*	HM ^2^	Neonatal	HCM ^4^
19	Male	*PTPN11*	None	N/A ^3^	None
20	Male	*PTPN11*	HM ^2^	Neonatal	HCM ^4^
21	Female	*PTPN11*	Fatigue	2 months	PVS ^5^
22	Male	*RAF1*	HM ^2^	Neonatal	PVS ^5^
23	Male	*RAF1*	HM ^2^	14 years	HCM ^4^
24	Female	*LZTR1*	HM ^2^	Neonatal	ASD ^7^
25	Male	*RIT1*	Arrythmia	Neonatal	PVS ^5^, ASD ^7^

^1^ CV S/S—cardiovascular signs and symptoms; ^2^ heart murmur; ^3^ N/A—not applicable; ^4^ HCM—hypertrophic cardiomyopathy; ^5^ PVS—pulmonary valve stenosis; ^6^ VSD—ventricular septal defect; ^7^ ASD—atrial septal defect.

**Table 2:** Echocardiography findings at the time of diagnosis.

	Diameter	Detroit Z Score
Interventricular septum	6.7 (IQR 5–9.75)	1.9 (1.7–5.2)
Posterior wall	4.5 (IQR 4–6.5)	−3.6 (IQR −4.3–1.5)
End diastolic diameter	23 (IQR 16–34.3)	−1.3 (IQR −2.5–−0.3)
Aortic valve	10 (IQR 8–14)	
	**Pressure gradient (mmHg)**	
Pulmonary artery	35 (IQR 18.5–53.5)	
Tricuspid valve	20 (IQR 15–35)	
Aortic valve	25 (IQR 23–75)	

**Table 3:** Summary of the most common CVS manifestations and affected genes.

Genes Affected	Total Patients	PVS ^1^ (%)	HCM ^2^ (%)
*PTPN11*	12	60.00	33.3
*RAF1*	5	0.0	41.66
Other Genes	8	40.0	25.0
Overall	25	40.0	48.0

^1^ PVS—pulmonary valve stenosis; ^2^ HCM—hypertrophic cardiomyopathy.

**Table 4:** Follow-up echocardiography findings.

	Diameter	Detroit Z Score
Interventricular septum	8 (IQR 6–12)	1.6 (0.2–3.2)
Posterior wall	6.5 (IQR 4–6.5)	−1.3. (IQR −4.4–2.1)
End diastolic diameter	32 (IQR 16–34.3)	−1.5 (IQR −2.5–−0.05)
Aortic valve	10 (IQR 8–14)	
	**Pressure gradient (mmHg)**	
Pulmonary artery	29 (IQR 20–38)	
Tricuspid valve	48 (IQR 45–48)	
Aortic valve	50 (IQR 17–50)	
Mitral valve insufficiency (+)	0.5 (IQR 0.5–2.0)	

**Table 5:** Frequency of neonatal cardiovascular manifestations (CVMs) by Noonan syndrome type.

NS Type	Affected Genes	Neonatal CVMs	Total Patients
Type 1	*PTPN11*	41.2%	12
Type 5	*RAF1*	44.4%	5
Other Types	Various	25.0%	8

**Table 6:** Comparative analysis of clinical features by Noonan syndrome types.

NS Type	Affected Gene	HCM ^1^	PVS ^2^	Developmental Delay	Stature Below −2 SD	Additional Symptoms
Type 1	*PTPN11*	33.3%	60.0%	50.0%	50.0%	60.0%
Type 5	*RAF1*	41.7%	0.0%	20.0%	40.0%	20.0%
Other Types	Various	25.0%	40.0%	30.0%	37.5%	30.0%

^1^ HCM—hypertrophic cardiomyopathy; ^2^ PVS—pulmonary valve stenosis.

## Data Availability

The data presented in this study are only available on request from the corresponding author due to privacy or ethical restrictions.

## Supplementary Materials

The following supporting information can be downloaded at https://www.mdpi.com/article/10.3390/genes15111463/s1, Table S1: Key Genetic Features, Clinical Manifestations and Cardiology Findings.

## References

[1] Tartaglia M., Gelb B.D., Zenker M. (2011). Noonan syndrome and clinically related disorders. Best Pract. Res. Clin. Endocrinol. Metab..

[2] Roberts A.E., Allanson J.E., Tartaglia M., Gelb B.D. (2013). Noonan syndrome. Lancet.

[3] Miller B.S. (2019). The History of Noonan Syndrome. Pediatr. Endocrinol. Rev..

[4] Allanson J.E., Bohring A., Dörr H.G., Dufke A., Gillessen-Kaesbach G., Horn D., König R., Kratz C.P., Kutsche K., Pauli S. (2010). The face of Noonan syndrome: Does phenotype predict genotype. Am. J. Med. Genet. A.

[5] Bell J.M., Considine E.M., McCallen L.M., Chatfield K.C. (2021). The Prevalence of Noonan Spectrum Disorders in Pediatric Patients with Pulmonary Valve Stenosis. J. Pediatr..

[6] Linglart L., Gelb B.D. (2020). Congenital heart defects in Noonan syndrome: Diagnosis, management, and treatment. Am. J. Med. Genet. C Semin. Med. Genet..

[7] Lee S.T., Ki C.S., Lee H.J. (2007). Mutation analysis of the genes involved in the Ras-mitogen-activated protein kinase (MAPK) pathway in Korean patients with Noonan syndrome. Clin. Genet..

[8] Baldo F., Fachin A., Da Re B., Rubinato E., Bobbo M., Barbi E. (2022). New insights on Noonan syndrome’s clinical phenotype: A single center retrospective study. BMC Pediatr..

[9] Zenker M., Edouard T., Blair J.C., Cappa M. (2022). Noonan syndrome: Improving recognition and diagnosis. Arch. Dis. Child..

[10] Jindal G.A., Goyal Y., Burdine R.D., Rauen K.A., Shvartsman S.Y. (2015). RASopathies: Unraveling mechanisms with animal models. Dis. Model. Mech..

[11] Fattah M., Raman M.M., Reiss A.L., Green T. (2021). PTPN11 Mutations in the Ras-MAPK Signaling Pathway Affect Human White Matter Microstructure. Cereb. Cortex.

[12] Athota L.P., Bhat M., Nampoothiri S., Gowrishankar K., Narayanachar S.G., Puttamallesh V., Farooque M.O., Shetty S. (2020). Molecular and clinical studies in 107 Noonan syndrome affected individuals with PTPN11 mutations. BMC Med. Genet..

[13] Tartaglia M., Zampino G., Gelb B.D. (2010). Noonan syndrome: Clinical aspects and molecular pathogenesis. Mol. Syndromol..

[14] Lepri F., De Luca A., Stella L., Rossi C., Baldassarre G., Pantaleoni F., Cordeddu V., Williams B.J., Dentici M.L., Caputo V. (2011). SOS1 mutations in Noonan syndrome: Molecular spectrum, structural insights on pathogenic effects, and genotype-phenotype correlations. Hum. Mutat..

[15] Riller Q., Rieux-Laucat F. (2021). RASopathies: From germline mutations to somatic and multigenic diseases. Biomed. J..

[16] Gelb B.D., Roberts A.E., Tartaglia M. (2015). Cardiomyopathies in Noonan syndrome and the other RASopathies. Prog. Pediatr. Cardiol..

[17] Karnik R., Geiger M. (2019). Cardiac Manifestations of Noonan Syndrome. Pediatr. Endocrinol. Rev..

[18] Gazzin A., Fornari F., Cardaropoli S., Carli D., Tartaglia M., Ferrero G.B., Mussa A. (2024). Exploring New Drug Repurposing Opportunities for MEK Inhibitors in RASopathies: A Comprehensive Review of Safety, Efficacy, and Future Perspectives of Trametinib and Selumetinib. Life.

[19] Hebron K.E., Hernandez E.R., Yohe M.E. (2022). The RASopathies: From pathogenetics to therapeutics. Dis. Model. Mech..

[20] Andelfinger G., Marquis C., Raboisson M.J., Théoret Y., Waldmüller S., Wiegand G., Gelb B.D., Zenker M., Delrue M.A., Hofbeck M. (2019). Hypertrophic Cardiomyopathy in Noonan Syndrome Treated by MEK-Inhibition. J. Am. Coll. Cardiol..

[21] Naik R.J., Bhangoo A. (2019). Chapter 3—Cardiac Manifestations in Noonan Syndrome: Effects of Growth Hormone Therapy. Noonan Syndrome.

[22] Sun L., Xie Y.M., Wang S.S., Zhang Z.W. (2022). Cardiovascular Abnormalities and Gene Mutations in Children With Noonan Syndrome. Front. Genet..

[23] Prendiville T.W., Gauvreau K., Tworog-Dube E., Patkin L., Kucherlapati R.S., Roberts A.E., Lacro R.V. (2014). Cardiovascular disease in Noonan syndrome. Arch. Dis. Child..

[24] Kaltenecker E., Schleihauf J., Meierhofer C., Shehu N., Mkrtchyan N., Hager A., Kühn A., Cleuziou J., Klingel K., Seidel H. (2019). Long-term outcomes of childhood onset Noonan compared to sarcomere hypertrophic cardiomyopathy. Cardiovasc. Diagn. Ther..

[25] Kiamanesh O., Greenway S.C., Dicke F., Ballantyne B., Mitrovic S., McGrath K., White J.A., Kent W.D.T., Andelfinger G. (2024). Treatment of RAF1-Related Obstructive Hypertrophic Cardiomyopathy by MEK Inhibition Using Trametinib. JACC Case Rep..

[26] Ommen S.R., Ho C.Y., Asif I.M., Balaji S., Burke M.A., Day S.M., Dearani J.A., Epps K.C., Evanovich L., Ferrari V.A. (2024). 2024 AHA/ACC/AMSSM/HRS/PACES/SCMR guideline for the management of hypertrophic cardiomyopathy: A report of the American Heart Association/American College of Cardiology Joint Committee on Clinical Practice Guidelines. J. Am. Coll. Cardiol..

[27] D’Onofrio G., Delrue M.A., Lortie A., Marquis C., Striano P., Jaworski M., Andelfinger G., Perreault S. (2023). Treatment of Refractory Epilepsy with MEK Inhibitor in Patients with RASopathy. Pediatr. Neurol..

[28] Li D., March M.E., Gutierrez-Uzquiza A., Kao C., Seiler C., Pinto E., Matsuoka L.S., Battig M.R., Bhoj E.J., Wenger T.L. (2019). ARAF recurrent mutation causes central conducting lymphatic anomaly treatable with a MEK inhibitor. Nat. Med..

[29] Lioncino M., Fusco A., Monda E., Colonna D., Sibilio M., Caiazza M., Magri D., Borrelli A.C., D’Onofrio B., Mazzella M.L. (2022). Severe Lymphatic Disorder and Multifocal Atrial Tachycardia Treated with Trametinib in a Patient with Noonan Syndrome and SOS1 Mutation. Genes.

[30] Leegaard A., Gregersen P.A., Nielsen T., Bjerre J.V., Handrup M.M. (2022). Succesful MEK-inhibition of severe hypertrophic cardiomyopathy in RIT1-related Noonan Syndrome. Eur. J. Med. Genet..

[31] Meisner J.K., Bradley D.J., Russell M.W. (2021). Molecular Management of Multifocal Atrial Tachycardia in Noonan’s Syndrome with MEK1/2 Inhibitor Trametinib. Circ. Genom. Precis. Med..

[32] Mussa A., Carli D., Giorgio E., Villar A.M., Cardaropoli S., Carbonara C., Campagnoli M.F., Galletto P., Palumbo M., Olivieri S. (2022). Mek inhibition in a newborn with raf1-associated noonan syndrome ameliorates hypertrophic cardiomyopathy but is insufficient to revert pulmonary vascular disease. Genes.

[33] Pierpont M.E., Basson C.T., Benson D.W., Gelb B.D., Giglia T.M., Goldmuntz E., McGee G., Sable C.A., Srivastava D., Webb C.L. (2007). Genetic basis for congenital heart defects: Current knowledge: A scientific statement from the American Heart Association Congenital Cardiac Defects Committee, Council on Cardiovascular Disease in the Young: Endorsed by the American Academy of Pediatrics. Circulation.

